# MEETING IN-PERSON NEEDS OF ELDERS LIVING IN RURAL COMMUNITIES BY UTILIZING APPS AND INTERNET ACCESS

**DOI:** 10.1093/geroni/igae098.0919

**Published:** 2024-12-31

**Authors:** Kate Clayton-Jones

**Affiliations:** FootCare by Nurses, Amherst, Massachusetts, United States

## Abstract

Challenges and success for meeting the in person needs of elders living in rural communities by utilizing apps and internet access takes tenacity and workarounds as many rural communities lack community wide internet, WiFi or cell phone coverage that providers of care can access. This lack of infrastructure impacts everything from first responder access and communication to efficient routing and access to information and charting by providers while in the home providing care. This lack of infrastructure is an impediment to quality care and also employment opportunities. Rural communities typically have more older adults residing in them. These older adults benefit from in home care, especially with ADLs. Technology access allows people to work from home, on the road and live in rural communities. It is a win win in health care when rural communities embrace community wide communication access.

